# Naturalness of Face-to-Face Medium and Video-Mediated Online Communication: Doubts About Evolutionary Mismatch

**DOI:** 10.3389/fsoc.2022.788447

**Published:** 2022-02-14

**Authors:** Yulia Shkurko

**Affiliations:** Department of Philosophy, Ulyanovsk State University, Ulyanovsk, Russia

**Keywords:** face-to-face communication, evolved adaptation, evolutionary mismatch, media naturalness theory, video-mediated communication, videoconferencing, computer-mediated communication, neurosociology

## Introduction

This study was motivated by doubts about the idea of the natural superiority of face-to-face medium, which develops both at the conceptual level (e.g., Kock, 2004, 2005, 2012) and is taken as a reference point when testing or developing improvements in technological communication systems (e.g., Almeida et al., 2012; Chua et al., 2012; Kegel et al., 2012; Kimura et al., 2020). The idea of natural superiority of face-to-face communication is complementary to evolutionary mismatch hypothesis, according to which the evolved human nature is less consistent with all other types of device-mediated communication, which can be a source of negative social and cognitive consequences (undermine immediate interpersonal interactions, impairment in the development of the ability for theory of mind and role-taking, etc.; e.g., Sbarra et al., 2019; Kalkhoff et al., 2020).

With regard to video-mediated online communication (VMOC), with an explicit or implicit acceptance of the idea of evolutionary mismatch, it is often concluded that imitation of face-to-face interaction through technical means or through users' learning and training is a valid way to improve communication (more on this below).

The purpose of the article is to encourage a discussion about whether video-mediated online communication should be brought closer to face-to-face communication or whether we should look for other ways, in particular: (i) the validity of a reference to the evolutionary past of humanity (at least in the meaning of the absence of an evolved biological mechanism for VMOC) in improving VMOC, (2) problematic nature of taking the idea of the natural superiority of face-to-face communication for granted, (3) the need for search for alternatives for the development of VMOC systems.

## The Naturalness of Face-to-Face Medium and the Diversified Nature of the Biological Communication Apparatus

The idea of the natural superiority of face-to-face communication underlies Kock's media naturalness hypothesis, which is quite popular in communication research (e.g., DeRosa et al., 2004; Simon, 2006; Vlahovic et al., 2012; Blau et al., 2017; Karl et al., 2021). According to this hypothesis, “the face-to-face medium is the most natural medium of all” (Kock, 2004, p. 124), arising from natural selection as the most efficient way of exchanging information in terms of the survival of our ancestors (Kock, 2004, 2005). Therefore, any forms of communication deviating from face-to-face interaction are considered as less suited to innate human communication capacities and as requiring greater cognitive efforts to exchange information, leads to an increasing communication ambiguity and a decreasing physiological arousal (Kock, 2004).

The media naturalness hypothesis is inherently complementary to the evolutionary mismatch hypothesis, central to evolutionary analysis (Lloyd et al., 2011) and fruitfully applied in different disciplines. According to the hypothesis, behavioral, mental and other adaptations, rigidly tied to the structure and functioning of the brain, which were useful for the survival of our ancestors in the past, may not be so in the new changed conditions. The evolutionary mismatch can be a source of negative social, psycho-emotional and other consequences; for example, affect the performance of organizations, lead to chronic psychological stress, be a source of low level of well-being, and so forth (Van Vugt and Ronay, 2013; Brenner et al., 2015; Kanazawa and Li, 2015; Li et al., 2017).

Kock proceeds from the fact that in the process of evolution our ancestors developed a biological apparatus tuned to the properties of face-to-face communication, such as colocation, synchronicity, facial expression, the ability to convey and observe body language, and speech language (Kock, 2005, p. 125; Kock, 2012, p. 386). Accordingly, there is a mismatch between the biological communication apparatus and the characteristics of modern means of communication. Fixing this evolutionary mismatch is used by Kock to argue the position about the need to approximate e-communications in business to the properties of natural face-to-face communication (Kock, 2005).

The statement that in the process of evolution humans have formed a single biological apparatus associated with face-to-face communication is insufficiently substantiated. Evidence from anthropology, evolutionary psychology and evolutionary sociology (e.g., Dunbar, 1997; Buss, 2019; Turner, 2021) suggests that our ancestors participated in communication, which (1) is characterized by varying degrees of representation of the above properties of face-to-face communication, (2) is connected with solving various adaptation problems. Accordingly, (3) neurobiological mechanisms associated with communication of various kind have evolved.

It is likely that our ancestors during scavenging or hunting (it is believed that our ancestors began to hunt since the time of Homo erectus, perhaps earlier; however, this point of view is not accepted by everyone, e.g., Nitecki and Nitecki, 1987) communicate with each other without being able to read non-verbal information, see the face of a fellow tribesman, and this kind of communication in an evolutionary sense is no less natural than face-to-face communication. In addition, by participating in face-to-face interactions, our ancestors solved various problems, for example, detecting social alliances, managing long-term pair-bonding, attaining and maintaining social rank, etc. (e.g., Kurzban et al., 2001; Fletcher et al., 2015; Maner and Case, 2016). Solving these adaptation problems can be considered in the context of maintaining strong or weak social ties and the corresponding development of cognitive abilities with the rewiring of the human brain (Turner and Maryanski, 2013; Turner, 2021).

As Turner and Maryanski (2013) demonstrated, originally to our ancestors (the last common ancestor to all the great apes who lived in the first half of the Neogene period until about 12–18 million years ago, when the orangutans split from other great apes) were characterized by weak social ties, low sociality, and organization (Turner, 2021). The changes took place about 5–6 million years ago, when solving the problem of survival in open areas of the African savanna led to the development of primary, first and second order emotions, large volume of neocortex, enhanced cognitive capacities, capacity for speech, capacity to produce and use cultural symbols that allowed them to become better organized (Turner, 2021, p. 239). Moreover, “at our genetic core, it is not likely that the ape in us disappeared; rather, it is more likely that new behavioral propensities were layered over the more ancient ways of behaving and organizing (...)” (Turner, 2021, p. 38). Other researchers also pay attention to the evolutionary layering of the modern human brain, for example, Dunbar when considering the evolution of language though transition of mankind from social grooming to telling of gossip (Dunbar, 1997, p. 61–62).

## Dysfunctional Effects of Approaching Video-Mediated Online Communication to the Properties of Face-to-Face Medium and Evolutionary Mismatch

One approach to the problem of improving VMOC is to approximate it to the properties of face-to-face interaction (see the references below). In the terms of evolutionary analysis, the implementation of this approach can be described as: (1) the desire to bring the environment (technologies) in line with the evolved biological communication apparatus associated with face-to-face medium, (2) humans' adaptation to a novel environment through learning, training and/or the process of habituating.

A technological strategy for simulating face-to-face communication in an online environment is implemented (or justified for subsequent implementation) by creating the illusion of physical co-presence (telepresence), tracking of the person's face, body language and gestures, improving the quality of video and sound, maximum synchronization, using Virtual Reality technology, system for eye contact, realization of the function of tactile sensations, neurostimulation, using Augmented Reality technologies, and so forth (e.g., Almeida et al., 2012; Chua et al., 2012; Kimura et al., 2020).

An adaptive approach to improving VMOC is expressed in recommendations for learning users certain rules of interaction through video-mediated online platforms (gaze direction, camera and microphone mute, voice volume, “hide self” view, using an external webcam, virtual proxemics rules, interactions to enhance the sense of group belongingness, and so forth; e.g., Bailenson, 2021; Bennett et al., 2021) and training in the use of VMOC systems (e.g., Rivet et al., 2021) to enable users to perform their social functions in a similar way to how it is done using a face-to-face medium. In addition, this approach postulates the possibility of achieving an optimum in social interactivity mediated by new technologies through self-organization (e.g., Paradisi et al., 2021).

The authors of the above and similar studies consider the lack of face-to-face communication properties in the online environment as the causes of the negative psychological and other consequences, decrease in the effectiveness of communication and/changes in the habitual performance of social roles. For example, non-verbal overload is considered as a cause of fatigue during long-term participation in videoconferences (Bailenson, 2021), the reduced visual and tactile/smell senses as a factor that can reduce the effectiveness of dance movement therapy (Paradisi et al., 2021), a low level of social presence (caused by incomplete view, lack of eye contact and life-size scaling, etc.) as a decrease in involvement in communication, mutual understanding and satisfaction with communication (Almeida et al., 2012; Chua et al., 2012; Kimura et al., 2020).

Without questioning that technological and learning/training ways of approaching VMOC to face-to-face interaction can improve the quality of communication, at least for some users, I would like to draw the attention of readers to the non-obviousness of considering the properties of face-to-face interaction as a guideline in this kind of search.

In the terms of evolutionary analysis (previous section) the technological strategy does not eliminate the evolutionary mismatch, but rather may exacerbate it. Evolutionary mismatch captures a moment of dysfunctional consequences as a result of mental and other human traits that were evolved in one environment being placed in another (Lloyd et al., 2011). The environment in the considered case should be understood as the social context in which communication was carried out to solve a certain adaptation problem.

Some of these dysfunctional effects are inherent in face-to-face communication environments and could potentially be eliminated in an online environment by keeping the VMOC properties in a standard form, for example, by maintaining the benefits of not having direct physical presence on cognitive and affective processes and by giving priority to speech (an idea of prioritizing the ability to convey and listen to speech over facial expressions, body language for the effectiveness of communication and proximity to face-to-face communication is also indicated in the media naturalness hypothesis in “speech imperative proposition,” Kock, 2004, p. 335). Spoken language as an adaptation for the transmission of information (Pinker, 1994/2007) is a later human evolutionary aacquisition than the cognitive abilities associated with reading and communicating non-verbal information (in particular, emotions; e.g., Turner and Maryanski, 2013; Turner, 2014)^1^. Saturation of video-online communication with non-verbal social cues through technical improvements can lead to the activation of more ancient mechanisms of social cognition and perception. These mechanisms may interfere with the implementation of the mechanisms associated with the speech aspect of communication, which is the most important in many areas of human activity. To illustrate, this can lead to an increase in forms of behavior inappropriate for the professional sphere, such as harassment, flirting, intrigue, aggression, etc. and interfering with work performance (e.g., Blackwell et al., 2019 indicates that embodiment and presence can intensify harassment in VR social reality).

Other consequences are specific to VMOC and not inherent in face-to-face communication in the traditional form; for example, the appearance of asymmetry in obtaining information about the interlocutor due to different technical means (AI-based emotion detection technologies, predictive technologies, automatic data capture and data analysis technologies, masking and deepfake technologies, etc.) that users have at their disposal, inequality and technological escalation associated with it. In addition, the technological and ideological legitimization of the idea of normativity and natural superiority of face-to-face communication may have negative consequences both for the development of the VMOC systems (we set limits in advance) and people (stress, anxiety, frustration, impaired well-being, etc.), since this ideal is unlikely to be achieved.

Thus, as follows from the above idea of the diversified nature of the biological communication apparatus and how the adaptive approach leads to this, VMOC generates its own sociality (see also works on the theory of social presence, Cui et al., 2013 for a review), which is (or becomes over time) no less natural than sociality associated with face-to-face medium. Therefore, the reference to the naturalness of face-to-face medium and evolutionary mismatch in the context of discussing the problems of modern VMOC systems requires at least concretization, depending on the goals and features of the users, allowing to identify specific evolved adaptations and adaptation problems. Otherwise, we face one-sided and potentially misleading interpretations of data indicating differences of face-to-face communication compared to VMOC, referring to our brain's unpreparedness for this kind of communication; for example, the results of such studies, which recorded partial activation of mirror neurons in video-mediated online circumstances (e.g., Dickerson et al., 2017), or data concerning other emotionality (intensity, valence, etc.) of video-mediated online compared to face-to-face communication in different categories of people (e.g., Riby et al., 2012; Schaarschmidt and Koehler, 2021).

## Instead of Conclusion: the Need to Study Alternative Ways to Improve Video-Mediated Online Communication

An alternative way is to move away from considering the problem of improving VMOC as a task to bridge the gap between VMOC and face-to-face medium. One approach involves focusing on the development of alternative institutions through the implementation of such VMOC systems that level the imperfections of social interactions such as excessive ritualization, particularism, prejudice, biased perceptions, etc. generated, among other things, by the properties of face-to-face communication. Orientation to the functional tasks of institutions, may help us to justify and accept the possibility for “non-natural” VMOC systems; for instance, neurotechnological invasive or non-invasive interventions in the human brain and other alternatives to enhancing cognitive abilities and customizing VMOC are worth exploring.

## Author Contributions

The author confirms being the sole contributor of this work and has approved it for publication.

## Conflict of Interest

The author declares that the research was conducted in the absence of any commercial or financial relationships that could be construed as a potential conflict of interest.

## Publisher's Note

All claims expressed in this article are solely those of the authors and do not necessarily represent those of their affiliated organizations, or those of the publisher, the editors and the reviewers. Any product that may be evaluated in this article, or claim that may be made by its manufacturer, is not guaranteed or endorsed by the publisher.

## Footnotes

^1^The capacity for speech arose about 2 million years ago, accompanied by the growth of the neocortex, while human emotional abilities developed 10–12 million years ago, associated with an increase in the subcortical areas (Turner, 2014).

## References

[B1] Almeida I. D. S. Oikawa M. A. Carres J. P. Miyazaki J. Kato H. Billinghurst M. (2012). “AR-based video-mediated communication: a social presence enhancing experience,” in 14th Symposium on Virtual and Augmented Reality (Rio de Janeiro). pp. 125–130. 10.1109/SVR.2012.427295638

[B2] Bailenson J. N. (2021). Nonverbal overload: a theoretical argument for the causes of zoom fatigue. Technol. Mind Behav. 2. 10.1037/tmb0000030

[B3] Bennett A. A. Campion E. D. Keeler K. R. Keener S. K. (2021). Videoconference fatigue? Exploring changes in fatigue after videoconference meetings during COVID-19. J. Appl. Psychol. 106, 330–344. 10.1037/apl0000906 33871270

[B4] Blackwell L. Ellison N. Elliott-Deflo N. Schwartz R. (2019). Harassment in social virtual reality: challenges for platform governance. Proc. ACM Hum. Comput. Interact. 3:100. 10.1145/3359202

[B5] Blau I. Weiser O. Eshet-Alkalai Y. (2017). How do medium naturalness and personality traits shape academic achievement and perceived learning? An experimental study of face-to-face and synchronous e-learning. Res. Learn. Technol. 25. 10.25304/rlt.v25.1974

[B6] Brenner S. L. Jones J. P. Rutanen-Whaley R. H. Parker W. Flinn M. V. Muehlenbein M. P. (2015). Evolutionary mismatch and chronic psychological stress. J. Evolut. Med. 30, 32–44. 10.4303/jem/235885

[B7] Buss D. M. (2019). Evolutionary Psychology. The New Science of the Mind. 6th Edn. New York, NY; London: Routledge.

[B8] Chua Y. Tee K. P. Yan R. Li L. Huang Z. (2012). “Towards more engaging telepresence by face tracking,” in Proceedings of the Workshop at SIGGRAPH Asia (WASA '12) (New York: Association for Computing Machinery), 137–141.

[B9] Cui G. Lockee B. Meng C. (2013). Building modern online social presence: a review of social presence theory and its instructional design implications for future trends. Educ. Inf. Technol. 18, 661–685. 10.1007/s10639-012-9192-1

[B10] DeRosa D. M. Hantula D. A. Kock N. D'Arcy J. (2004). Trust and leadership in virtual teamwork: a media naturalness perspective. Hum. Resour. Manag. 43, 219–232. 10.1002/hrm.2001625855820

[B11] Dickerson K. Gerhardstein P. Moser A. (2017). The role of the human mirror neuron system in supporting communication in a digital world. Front. Psychol. 8, 698. 10.3389/fpsyg.2017.00698 28553240 PMC5427119

[B12] Dunbar R. (1997). Grooming, Gossip and the Evolution of Language, 6th Edn. London: Faber and Faber Limited.

[B13] Fletcher G. J. O. Simpson J. A. Campbell L. Overall N. C. (2015). Pair-bonding, romantic love, and evolution: the curious case of homo sapiens. Perspect. Psychol. Sci. 10, 20–36. 10.1177/1745691614561683 25910380

[B14] Kalkhoff W. Serpe R. T. Pollock J. (2020). Is Video Chat a Sufficient Proxy for Face-to-Face Interaction? Biosociological Reflections on Life during the COVID-19. Available online at: https://thisviewoflife.com/is-video-chat-a-sufficient-proxy-for-face-to-face-interaction-biosociological-reflections-on-life-during-the-covid-19-pandemic/

[B15] Kanazawa S. Li N. P. (2015). Happiness in modern society: why intelligence and ethnic composition matter. J. Res. Pers. 59, 111–120. 10.1016/j.jrp.2015.06.004

[B16] Karl K. A. Peluchette J. V. Aghakhani N. (2021). Virtual work meetings during the COVID-19 pandemic: The good, bad, and ugly. Small Group Res. 10464964211015286. 10.1177/10464964211015286 PMC8165498 38603094

[B17] Kegel I. Cesar P. Jansen J. Bulterman D. Stevens T. Kort J.. (2012). “Enabling ‘togetherness' in high-quality domestic video conferencing,” in Proceedings of the 20th ACM international conference on Multimedia (MM '12). Association for Computing Machinery (New York, NY), 159–168.

[B18] Kimura S. Ooseki E. Aburakawa Y. Yamaguchi M. (2020). Evaluation and formulation of the sense of social telepresence in video-mediated communication systems: contribution of eye contact to enhancing social telepresence. J. Soc. Inf. Display 29, 1–17. 10.1002/jsid.97625855820

[B19] Kock N. (2004). The psychobiological model: towards a new theory of computer-mediated communication based on darwinian evolution. Organ. Sci. 15, 327–348. 10.1287/orsc.1040.0071

[B20] Kock N. (2005). Media Richness or Media Naturalness? The evolution of our biological communication apparatus and its influence on our behavior toward e-communication tools. IEEE Trans. Profess. Commun. 48, 117–130. 10.1109/TPC.2005.84964927295638

[B21] Kock N. (2012). “Media naturalness theory: human evolution and behaviour towards electronic communication technologies,” in Applied Evolutionary Psychology, ed S. Craig Roberts (Oxford: Oxford University Press).

[B22] Kurzban R. Tooby J. Cosmides L. (2001). Can race be erased? Coalitional computation and social categorization. Proc. Natl. Acad. Sci. U.S.A. 98, 15387–15392. 10.1073/pnas.251541498 11742078 PMC65039

[B23] Li N. P. van Vugt M. Colarelli S. M. (2017). The Evolutionary Mismatch Hypothesis: Implications for Psychological Science. Curr. Dir. Psychol. Sci. 27, 38–44. 10.1177/0963721417731378

[B24] Lloyd E. A. Wilson D. S. Sober E. (2011). Evolutionary Mismatch and What to Do About It: A Basic Tutorial. Available online at: https://evolution-institute.org/wp-content/uploads/2015/08/Mismatch-Sept-24-2011.pdf

[B25] Maner J. K. Case C. R. (2016). “Dominance and prestige: dual strategies for navigating social hierarchies,” in Advances in Experimental Social Psychology, Vol. 54, eds J. L. Olson and M. P. Zanna (London: Elsevier), 129–180.

[B26] Nitecki M. N. Nitecki D. V. (Eds.). (1987). The Evolution of Human Hunting. New York, NY; London: Plenum Press.

[B27] Paradisi P. Raglianti M. Sebastiani L. (2021). Online communication and body language. Front. Behav. Neurosci. 16:709365. 10.3389/fnbeh.2021.709365 34557076 PMC8452979

[B28] Pinker S. (1994/2007). The Language Instinct. New York, NY: Harper Perennial Modern Classics.

[B29] Riby D. M. Whittle L. Doherty-Sneddon G. (2012). Physiological reactivity to faces via live and video-mediated communication in typical and atypical development. J. Clin. Exp. Neuropsychol. 34, 385–395. 10.1080/13803395.2011.645019 22260255

[B30] Rivet E. B. Cholyway R. Edwards C. Wishnoff M. Raza O. Haynes S.. (2021). Video-mediated breaking bad news simulation. Clin. Teach. 18, 424–430. 10.1111/tct.13387 34101333

[B31] Sbarra D. A. Briskin J. L. Slatcher R. B. (2019). Smartphones and close relationships: the case for an evolutionary mismatch. Perspect. Psychol. Sci. 14, 596–618. 10.1177/1745691619826535 31002764

[B32] Schaarschmidt N. Koehler T. (2021). Experiencing emotions in video-mediated psychological counselling versus to face-to-face settings. Societies 11, 20. 10.3390/soc11010020

[B33] Simon A. F. (2006). Computer-mediated communication: task performance and satisfaction. J. Soc. Psychol. 146, 349–379. 10.3200/SOCP.146.3.349-379 16783986

[B34] Turner J. (2021). On Human Nature. The Biology and Sociology of What Made Us Human. New York, NY: Routledge.

[B35] Turner J. H. (2014). “The evolution of human emotions,” in Handbook of the Sociology of Emotions. Vol. II. Handbooks of Sociology and Social Research, eds J. E. Stets and J. H. Turner (Dordrecht: Springer Science+Business Media), 11–31.

[B36] Turner J. H. Maryanski A. (2013). “The evolution of the neurological basis of human sociality,” in Handbook of Neurosociology, eds D. D. Franks and J. H. Turner (New York, NY; London: Springer Science+Business Media B.V.).

[B37] Van Vugt M. Ronay R. (2013). The evolutionary psychology of leadership: theory, review, and roadmap. Organ. Psychol. Rev. 4, 74–95. 10.1177/2041386613493635

[B38] Vlahovic T. A. Roberts S. Dunbar R. (2012). Effects of duration and laughter on subjective happiness within different modes of communication. J. Comput. Mediated Commun. 17, 436–45. 10.1111/j.1083-6101.2012.01584.x

